# Mele’s Digital Zygote: Developer Responsibility for Neural Networks

**DOI:** 10.1007/s11948-025-00566-9

**Published:** 2025-11-26

**Authors:** Anders Søgaard, Filippos Stamatiou

**Affiliations:** 1 Department of Computer Science, University of Copenhagen, København, Denmark; 2 Department of Communication, University of Copenhagen & Department of Philosophy, Stellenbosch University, Stellenbosch, South Africa

**Keywords:** Ethics of AI, Moral responsibility, Responsibility gaps, Developer responsibility, Accountability

## Abstract

Should developers be held responsible for the predictions of their neural networks—and if not, does that introduce a responsibility gap? The claim that neural networks introduce a responsibility gap has seen significant pushback, with philosophers arguing that the gap can be bridged, or did not exist in the first place. We show how the responsibility gap turns on *whether we can distinguish between foreseeable and unforeseeable* neural network predictions. Empirical facts about neural networks tell us we cannot, which seems to force developers to either assume full responsibility or no responsibility at all, introducing a responsibility gap—unless, of course, the same empirical facts hold true of humans, in which case there is no gap, but the trouble is simply with the classical notion of responsibility. We revisit and revise Mele’s Zygote, as well as the famous Palsgraf case, and argue that in fact, what complicates responsibility assignment for neural networks also complicates responsibility assignment for humans, and humans seem to confront us with the same all-or-nothing dilemma. Thus, we agree there is no technology-induced responsibility gap (there was no gap in the first place), but for slightly different reasons than our predecessors.

**Our Argument** Should developers be held responsible for blameworthy predictions from their neural networks? Reviewing arguments for and against developer responsibility, we conclude that the question turns on whether we can decide what predictions were foreseeable. From a small set of uncontroversial empirical facts ($f_1-f_5$) about neural networks, we conclude that it is *not* possible to differentiate between foreseeable and unforeseeable predictions. On standard accounts, this creates a *responsibility gap* if and only if the foreseeability of the consequences of neural network inferences is different from the foreseeability of the consequences of our own actions. Since humans also satisfy $f_1-f_5$, we argue that in the human case, it is also impossible to differentiate between foreseeable and unforeseeable actions (not to speak of their consequences). So, technically speaking, there is no responsibility gap.

## Responsibility for Neural Networks

The rapid introduction of neural networks into our daily activities has led many to ask who is responsible when such networks cause harmful outcomes (Matthias, 2004; Strasser, 2021, Tigard, 2021b; Nyholm, 2023). We focus on who is responsible for outcomes caused by *neural network inferences*. Say, for example, a neural network spam filter makes a harmful prediction – in this case, a false positive:

### Example 1

A neural network is used for detecting spam emails. The positive result of an HIV test of your only child is sent over email, but caught in your spam filter. Not knowing the results, you fail to initiate treatment, and your child dies. Who, then, would be responsible?

Or say that a neural fake news detection tool introduces similar false positives:

### Example 2

A neural network is used for fake news detection and mistakenly blocks the story that a presidential candidate paid hush money to an adult movie actor. The story could have changed the election, but did not – as a direct result of the error-prone detection tool. Who, then, would be responsible?

Spam filters and fake news detection tools are generally not considered high-risk applications, e.g., in the EU’s AI Act, and are typically allowed to run without a human in the loop. However, they can easily lead to harm, and it remains unclear *who* then is to blame? Some possible parties who may bear responsibility include:

(i) The developers who created and trained the neural network. Developers may arguably have a responsibility to ensure that the network is designed and trained in a way that *minimizes bias, errors, and the risk of harmful outcomes*.
(ii) The organizations or individuals that deploy the neural network: They may arguably have a responsibility to ensure that the network is used in an appropriate and ethical manner and that any potential negative impacts are identified and addressed.
(iii) The regulators and policymakers: They may have a responsibility to establish guidelines and regulations that ensure neural networks are used in a safe and ethical manner, or to hold organizations accountable when they fail to do so.

The responsibility for harmful predictions made by neural networks need not be exclusive to one party but could be spread among multiple parties involved in the development, deployment, and regulation of these systems (Nyholm, 2018; Strasser, 2021). We ignore this complexity and focus on the developer’s responsibility, but many of our arguments generalize to organizations and regulators as well. We also ignore strict liability and intentional harms; see, e.g., Sparrow (2007), for a discussion of questions around responsibility for neural network predictions in the context of intentional harms.

Matthias (2004) famously argued that due to the complexity and unforeseeability of their inferences, neural networks introduce a *responsibility gap*. Recent work has elaborated on the epistemic implications of such a gap (Coeckelbergh, 2020; Veluwenkamp, 2025), and how appeal to traditional concepts in moral philosophy, e.g., such as *reactive attitudes* (Oimann & Tollon, 2025), can side-step the gap. The fundamental question remains: How can one be responsible for something that is unforeseeable? The idea that responsibility turns on foreseeability, is common in folk psychology (Markman & Tetlock, 2000) and moral philosophy (Miller, 2017). We also share this premise with Matthias (2004), i.e., the idea that your responsibility for harm depends in part on its foreseeability to you. This is also consistent with the idea that responsibility turns on intent (Dworkin, 1987) and being conscious (Levy, 2014). Strictly speaking, things are rarely foreseeable, but we take an event $e$ being foreseeable to mean that most humans will assign $e$ high probability given the current conditions. The event being foreseeable to someone is thus a necessary requirement for them being responsible for it. This is obviously not a sufficient requirement. Intent may, for example, play an important role. Many take consciousness to be a prerequisite for foreseeability, but we ignore this discussion for now. Here we are merely concerned with the problem of assigning responsibility in cases where events are *not* foreseeable.

Various authors have pushed back against the idea of a responsibility gap, claiming the gap can be bridged or mitigated (Nyholm, 2018; Tigard, 2021a; Tollon, 2023; Vallor & Vierkant, 2024; Oimann & Tollon, 2025), reduced to other problems (Llorca Albareda, 2025), or that it is exaggerated or illusory (Tigard, 2021b, Königs, 2022, Hindriks & Veluwenkamp, 2023, Kiener, 2025). Our contribution to this literature is as follows: We show how the responsibility gap discussion turns on (our ability to decide) the foreseeability of predictions, and how foreseeability relates to Mele’s Zygote (Alfred, 2006) and the 1928 legal case of Palsgraf v. Long Island Railroad. Our move is thus similar to that of (Oimann & Tollon, 2025), taking a step to explore how the gap relates to older discussions in moral philosophy. This leads us to present an argument that the responsibility gap is illusory but for reasons different from those previously discussed in the literature.

### Neural Networks

First, let us consider the relevant empirical facts about how neural networks are trained:

- $f_1$ Neural networks are initialized randomly, by setting their (millions or billions of) parameters to random numbers.
- $f_2$ From then on, randomly sampled batches of data are presented to the network, one batch at a time, and the network is then adjusted according to the gradients of its loss (error) on each batch.
- $f_3$ The training procedure does not converge to a global optimum.
- $f_4$ For some networks, the number of local optima is exponential in the numberof parameters (Auer et al., 1995).

These four facts about neural networks will be important for our arguments below, and have an important corollary, which is the main premise of the literature on explainable artificial intelligence:^1^

modern deep learning methods lack […] algorithmic transparency. While the heuristic optimization procedures for neural networks are demonstrably powerful, we don’t understand how they work, and at present cannot guarantee *a priori* that they will work on new problems. (Lipton, 2018)

In sum, neural networks are randomly initialized, then optimized on random batches of data, and the end result is one out of possibly billions of locally optimal models that are all too complex for humans to understand. Combined with the open-endedness and drift of the data (call this fact $f_5$), this makes neural network predictions unforeseeable in a deep sense. This means developers may not be able to predict negative impacts of the neural network, and therefore, they should perhaps not be held fully responsible. It is important to appreciate this consequence of $f_1$–$f_4$: Since neural networks are not causally determined (closed) by the training data ($f_1$) – nor by any other set of easy-to-understand factors – and since the training procedure is *not* *a priori* predictable.

This observation motivates *The All-or-Nothing Assumption* introduced in $\S2$. Whether or not it introduces a responsibility gap is the main question motivating our work. A responsibility gap normally means that we are confronted with harms for which no responsibility can be assigned. Of course this is relative to our ability to normally assign responsibility. In other words, neural networks introduce a responsibility gap if assigning responsibility for harms induced by such networks, is harder than assigning responsibility to humans.

## Arguments for Blaming Developers

A common argument *for* blaming developers for blameworthy decisions focuses on negligence. Let us call it The Negligence Argument. It goes as follows. Developers have control over the design and training of the neural network: They have the knowledge and expertise to design and train the network in a way that minimizes the risk of bias, errors, and harmful outcomes. Therefore, when the network makes a foreseeable *and* blameworthy decision, the developers are responsible for not taking the necessary precautions to prevent it, that is, for having acted negligently (Sarch & Abbott, 2019). Königs (2022) has argued that responsibility gaps should fulfill a *plausibility constraint*, according to which responsibility gaps arise only in situations where the harmful outcome is not the result of carelessness or malice. With that in mind, negligent behavior by developers does not give rise to responsibility gaps, as the responsibility remains with the developer. However, not how The Negligence Argument turns on foreseeability, a quality of neural networks apparently negated by $f_1$–$f_4$.

It is also possible to provide arguments for blaming developers that do not turn on foreseeability. If there were accepted standards for training and evaluating neural networks, developers that do not live up to these standards, would be blameworthy (Lechterman, 2023). The trouble, of course, is that following best practice or community-wide standards does not prevent harm. It may reduce the risk of harm, but in the context of neural networks with millions or billions of parameters, it is demonstrably impossible to set up perfect guard rails (Wolf et al., 2023). Therefore, the responsibility gap, if one exists, does not depend on the quality of the standards; it is *not* poor standards that create responsibility gaps. If a responsibility gap is introduced, when harm is possible for which blame cannot be assigned, weakening the above arguments will not dissolve it. Complex, probabilistic models will always have a non-zero probability of creating harmful predictions, recommendations or content (Wolf et al., 2023). So, regardless of how good standards we adopt, it seems there will always be space for misalignments that give rise to responsibility gaps (Veluwenkamp & Hindriks, 2024). Failure to comply with standards can be blameworthy as such. Complying with standards does not eliminate risk of harm. If such harm is predictable to developers, their actions may still be blameworthy.

Another type of argument would focus on how developers, in spite of the opacity of neural networks, are financially or professionally rewarded for their successful deployment. The argument would go like this: If developers benefit from their positive consequences, they should also be held responsible for their negative consequences. This is simply the developer’s gamble: A developer may accept unpredictable blame, because financial or professional rewards are significant and more likely than blame. This argument seems to make developers responsible for all neural network inferences, given that they are potentially rewarded for *all* their network’s inferences. Let us call this argument The Flipside Argument.

The Flipside Argument is motivated by and reflects the stark difference between blame and praise. As Anderson et al. (2020) has argued, empirical evidence shows that people are more interested in causality and the severity of outcomes when assigning blame rather than praise. Blame helps us signal our good moral character, while praise helps us establish and maintain good relationships with others. Apart from their functional differences, blame and praise also exhibit a well-documented asymmetry. For instance, one is more likely to be blamed for bad outcomes than be praised for good outcomes (Hindriks, 2008). This asymmetry seems to extend to current AI technology, including LLMs and generative AI (Porsdam Mann et al., 2023; Nyholm, 2025).

Most theories of moral responsibility focus on blame and blameworthiness rather than on praise and praiseworthiness. This demonstrates the importance of *getting it right* when we blame others. Wrongfully praising someone rarely causes harm. Wrongfully blaming someone, however, typically involves inflicting unnecessary harm. The asymmetry between blame and praise reflects their difference in relative importance for both ethical theory and everyday moral practice.

This leaves us with the following dilemma: Either (i) developers are responsible for *all* decisions made by their neural network; or (ii) they are not responsible at all. The Flipside Argument speaks to (i); The Negligence Argument normally speaks to (i), but combined with the empirical facts about neural networks, it speaks to (ii). We call the dilemma *the All-or-Nothing Assumption*. The argument for this assumption is that in so far as responsibility is grounded in concerns about harm, and harm is unforeseeable, there is no principled way of dividing neural network principles into those that are relevant for moral responsibility, and those that are not. A developer would have to be responsible always or never.

## Arguments Against Blaming Developers

There are at least two standard arguments *against* blaming developers for blameworthy decisions made by neural networks. Let us call the first The Complexity Argument. It runs like this: Neural networks are complex systems. The decisions made by a neural network can be influenced by a wide range of factors, including the data it was trained on, the design of the network, and the specific input it is given. This complexity makes it difficult to identify a single point of failure or a single party that is responsible for a blameworthy decision. It is important for The Complexity Argument to be philosophically significant that the complexity of artificial intelligence is such that other skilled developers would also not be in a position to identify such points of failure (Matthias, 2004). The Complexity Argument thus turns on the opacity of neural networks (Rudin, 2019).

The second argument is a bit different. Let us call it The Control Argument: Developers may not have complete control over the use of the neural network: Once a neural network is deployed, it may be used in ways that the developers did not anticipate or intend. This can make it difficult to hold the developers fully responsible for any negative consequences that result.^2^

Since complexity on its own does not necessarily lead to opacity and unforeseeability, we add a third argument to make our concerns explicit – The Unforeseeability Argument, motivated by the unforeseeability of neural network inferences. This argument sits between The Complexity Argument and The Control Argument. It emphasizes complexity, but of the network itself, not the dynamics around its external factors. Complexity means reduced predictability, even when the network is used as intended. The unforeseeability of future predictions is thus not a result of limited control over how the neural network is used. It is also not a result of the complexity of the context in which the neural network is put to use. It is a result of the complexity, size, and randomness of the network itself.

## Is the Responsibility Gap Illusory?

Several authors have suggested that responsibility gaps are illusory (Hindriks & Veluwenkamp, 2023; Kiener, 2025, Llorca; Albareda, 2025), reducible to other problems (Llorca Albareda, 2025; MirzaeiGhazi & Stenseke, 2025), or that they do not undermine current moral responsibility practices (Königs, 2022, Tollon, 2023; Vallor & Vierkant, 2024). Here, we focus on Tigard (2021b) who argues that the challenges introduced by neural networks can be met relying on our standard toolbox of moral practices. Tigard writes:

[…] the potential normative mismatch in accountability can be repaired indirectly by retrospective measures toward human associates (designers, programmers, or users) or directly by adopting forward-looking aims in our interactions with technology. (Tigard, 2021b, p. 604)

Retrospective measures against designers are possible, but how do we ensure that they track harm that can be attributed to the neural network, given the empirical facts $f_1$–$f_4$? Similarly, while (forward-looking) debugging is an appropriate response to the malfunctioning of a neural network, it does not regulate the risk of developers releasing products that are not safe for use, given the empirical facts $f_1$–$f_4$. Tigard (2021b) seems to imply that the empirical facts, i.e., the opacity of neural networks, is no reason *not* to make developers responsible:

[…] fully functional adult human beings also fail to provide adequate reasons for their decisions, say, due to implicit biases […] and this has not stopped us from demanding answers from them.

Tigard talks about providing adequate reasons for the decisions made. This is a way of introducing the idea of foreseeability. The decisions of someone are foreseeable if we can give adequate reasons for them, regardless of whether we adopt a first-person or third-person perspective. On most accounts of foreseeability, being able to give adequate reasons for $e$ is also the only way to robustly predict $e$. Tigard, in effect, argues that there is no responsibility gap because the decisions of humans are unforeseeable, yet blameworthy. One common response to such a position is to argue that while humans *sometimes* fail to provide adequate reasons for their decisions, they are not *in principle* limited in the same way neural networks are. We believe that, if anything, the opposite is true. Given current technologies, humans are inherently black-boxed, to the extent that our decisions are determined by elusive neural activity. This is the case both from a first-person and third-person perspective. Neural network inferences can be printed out and they are, in principle, glass boxes. Their inner workings are complex, and arguably too complex to fathom, but everything happens in plain sight. Humans are more opaque than neural networks. Some consequences of *our* actions are also unforeseeable to ourselves. The question is if we can, in advance, tell these actions apart from the actions whose consequences we can predict? This is what negligence turns on. The question becomes

*whether we are less capable when it comes to neural networks (compared to humans) to decide which consequences were foreseeable?*

If the answer to this question is yes, a responsibility gap exists. If the answer is no, there is no such gap. We saw in our motivation of *The All-or-Nothing Assumption* that the distinction between foreseeable and non-foreseeable consequences is not feasible for neural networks. This means that there is a responsibility gap unless the same holds for humans. Does a similar all-or-nothing assumption hold true for known moral dilemmas involving humans? If we can decide which consequences of our past actions were foreseeable, we can make a meaningful distinction between the consequences of our actions and neural network inferences, introducing a qualitatively different responsibility assignment problem for neural networks. Because we cannot decide which neural network inferences were foreseeable, and therefore cannot make judgments about negligence, we have a (relative) responsibility gap for neural networks. If, however, a similar all-or-nothing assumption can be made for humans, there is no gap, no relevant difference between our actions and neural network inferences. Selective responsibility assignment is impossible, and for that reason there is no responsibility gap. To consider this last possibility, we turn to two famous cases from the moral responsibility literature from philosophy and law.

## Mele’s Zygote

To see whether there is a responsibility gap after all, we need to compare our intuitions about developers of neural networks to more traditional moral dilemmas. Here, we focus on a famous thought experiment proposed by Alfred Mele, commonly known as The Zygote Argument (Alfred, 2006).^3^ We turn to the Zygote Argument, as well as the so-called Palsgraf case in legal responsibility theory, to compare the question of developer responsibility for neural networks to related questions about human blameworthiness, and to discuss whether the unforeseeability of neural network inferences is relevantly different from the unforeseeability of the consequences of our actions.

In the Zygote Argument, Diana – a protagonist with perfect knowledge of the laws of nature and the state of the entire universe at the time, and the ability to manipulate biological matter as she sees fit – creates a zygote in Mary with the intention of causing a certain event E to occur thirty years later. Diana knows that the zygote will develop into an ideally self-controlled agent who will make a decision based on rational deliberation, and that this decision will bring about event E. Mele used the strong intuition that Ernie, the person who develops from the zygote, cannot be held responsible for bringing about E.^4^ Our interest lies with the intuition that the blameworthy one is Diana.

Now, what does this intuition have to do with developer responsibility for blameworthy decisions made by neural networks? If Diana were a developer designing good old-fashioned symbolic artificial intelligence, capable of bringing about E and she did in fact bring about E, she would also be responsible for bringing about E. But what if E was a non-intended side-effect of training and releasing a neural network? This seems more comparable to a thought experiment in which Diana is Ernie’s mother engaging in parenting practices that happen to bring E about.

## The Digital Zygote

Suppose we replace Diana with a computer programmer and Ernie with a neural network of about 80 billion parameters, to see how that changes our intuitions, if at all. Specifically, we will be interested in whether developers of neural networks are responsible for

bad effects resulting from something that is done with good intentions. To what extent should AI experts be responsible for preventing unintended, undesirable consequences of AI technology that functions exactly as it is supposed to do? (Hedlund & Persson, 2022)

Here is our revised thought experiment. For our purposes, we turn Diana into a regular, non-omniscient computer programmer.

**The Digital Zygote.** Diana trains a neural network Z. She fits Z’s 80 billion weights as she does because she wants a certain event E to occur (directly caused by Z’s prediction that $y$) in some distant future with a non-zero probability. Given her knowledge of the input data distribution, she knows that with high probability the network will one day, sooner or later, be presented with input data bringing about E. Thirty years later, Z is presented with this input data and brings about E.

On the face of it, the updated version of the thought experiment no longer seems to get off the ground. Why not? Because Diana, given $f_1$–$f_5$, cannot generally predict whether E will be brought about with a small, but non-zero probability. This holds for both static and drifting data distributions. In other words, we need Diana to keep some of her abnormal powers to stay responsible for Z’s bringing about E. She does not have to remain omniscient, but she needs to be able to compute a distribution of possible inferences through 80 billion parameters. If the data distribution drifts, she would have to be able to predict this drift, too.

The argument for maintaining Diana’s moral responsibility, in the case of a static input distribution (for simplicity), would run:

- ($p_1$) Diana can compute the posterior distribution of the neural network she trains.
- ($p_2$) Diana has an intention for the neural network to bring about E.
- ($p_3$) If Diana has the intention for the neural network to bring about E and can, in general, compute its posterior distribution, then she is capable of bringing about E.
- $\rightarrow$ Diana is responsible for bringing about E.

Now, notice what happens if $p_1$ does not hold, then the *modus ponens* inference no longer applies. Obviously, this does not mean that Diana is *not* responsible for bringing about E, but it means her responsibility becomes an open question. If $\neg p_1$, $p_2$ and $p_3$, we cannot conclude anything about the moral responsibility of Diana for E. The same holds if If $\neg p_1$, $\neg p_2$ and $p_3$, of course. Note, though, how it also follows that if $p_1$, and Diana intends *not* to bring about E, she is possibly still responsible for E, because if E happens, Diana has been negligent. However, her responsibility always seems to depend on $p_1$. Or does it? In general, developers cannot foresee the predictions of the neural networks they train, *qua* the Unforeseeability Argument. But can we nevertheless assign responsibility? Here are some options:

(i) The developer is, in spite of the unforeseeability of E, nevertheless responsible for the predictions of the neural networks, and therefore for E.
(ii) The developer is *not* responsible for the predictions of the neural networks, and hence not for E.
(iii) The developer is not responsible for the predictions of the neural networks, but for enabling the predictions of the neural networks to impact end users in the wild.

Option (i) is found in recent policy proposals.^5^ It is the most widely held view, but seems inconsistent with the common assumption that moral responsibility tracks control, or some version of *could-have-done-otherwise*. The question of whether a responsibility gap exists, comes down to whether moral responsibility ever tracked control in the first place. It is clear from our revised version of Mele’s Zygote Argument that in the absence of control, i.e., Diana’s ability to compute the posterior distribution, the argument for her responsibility would be unsuccessful. Given $f_1$–$f_5$, the developer has little control over the future predictions of the neural networks. The only way in which the developer thus could have done otherwise, would be by *not* training the neural network in the first place or by *not* releasing it in the wild.

The situation is somewhat analogous to a parent who does not take full responsibility for their child later in life (after the product release date). Option (ii) would mean that some other responsible person must be identified, unless no one is made responsible. Option (iii) may look like a softening of (i), but is in fact stronger, given that it does not make causing harm a necessary condition for blame-worthiness. It will not buy us anything to try to distinguish between foreseeable and unforeseeable decisions, since all decisions are unforeseeable by $f_1$–$f_5$, i.e., under The All-or-Nothing Assumption. If the same does *not* hold for the consequences of *our* actions, Matthias (2004) and the responsibility gap hypothesis would be vindicated.

Strictly speaking, the consequences of *our* actions also depend on the complex interaction of about 80 billion parameters. In fact, it seems that $f_1$–$f_5$ are true for humans, too. They are initialized randomly ($f_1$), since genetic drift is random (Chen et al., 2017). Humans are exposed to stimuli in unpredictable order ($f_2$). We have no reason to believe that we converge to a global optimum ($f_3$), and we can at any time find ourselves in a lot of possible states ($f_4$). Hence, our actions are, strictly speaking, equally unforeseeable. Hence, it seems that at this point we have no reason to postulate a responsibility gap – even if we have good reasons to disagree with Tigard (2021b) on the details.

## Pragmatic Responsibility?

The All-or-Nothing Thesis seems like a Gordian knot leaving us only with two radical options: (a) to give up on accountability completely, or (b) to accept full developer responsibility for any future neural network inference. But would it not be a solution to simply do away with responsibility entirely and instead adopt a more pragmatic position around risk management? After all, our main interest in responsibility assignment is forward-looking, namely to prevent future harm. Classical responsibility and risk management have very different profiles, though. Risk management is pragmatic and shifts the focus from the developer’s causal role to the developer’s likely causal role in future harms.

If developers of neural networks by definition cannot predict the actions of the networks *ex ante*, they seem exempt from responsibility, leading to an apparent responsibility gap – unless humans are generally exempt from responsibility. Now we can steer clear of the problem altogether, it seems, if we put risk management rather than responsibility assignment center stage. Libertarians, compatibilists, and physicalists agree that we have *pragmatic* reasons for punishment or quarantining protagonists that cause significant danger or harm. Some forms of punishment are highly controversial, but preventing a school shooter from entering a school with an assault riffle seems like the kind of thing we would want to do. In this case, we punish or quarantine the shooter–not their parents, their teachers, or their physicians–because it is the most effective way to prevent harm. We do not need to decide if the shooter is truly responsible for their actions, in order to decide that we need to stop them from killing innocent children. Even a shooter under the influence of a vicious brain tumor or manipulated by an external controller should be stopped.

Let us say that someone who we feel obliged to contain or restrain to prevent serious harm, is $p$-responsible in virtue of constituting a very probable risk. That is:

$p$**-responsibility:** An agent A is $p$-responsible for an event E if containing or restraining A is the most effective way to prevent E from happening. Being $p$-responsible does not entail being morally responsible; nor vice versa.

What guidance does $p$-responsibility give us in the case of spam filters or fake news detection? Clearly, a harmful spam filter or an overly aggressive fake news monitoring system can be taken off the market. We may not always know if a system is harmful in advance, but when we learn so, we can retroactively remove it. Of course the developer can also be restrained somehow. Moreover, we can gate-keep the market for neural networks by requiring product-scale neural networks to pass tests designed to evaluate their short- and long-term risks; as well as by requiring developers to pass tests designed to evaluate the risk of them producing neural networks that will eventually cause harm.

When would we restrain developers? What would be a reason to restrain the developer rather than simply take their products off the market? If we have reason to believe the developer will be likely to release more products with harmful effects, we may want to restrain the developer. If, for example, we had evidence that the developer *on purpose* optimized for blameworthy decisions, this would be strong reason to think restraining the developer would be the most effective way to prevent the release of such products in the future. If the developer had a track record of releasing such products, intended or not, this would perhaps be another reason to restrain the developer. Otherwise, it seems that taking the product off the market would be the most effective way of preventing such harms.

Note how the definition of $p$-responsibility does not constitute a moral theory. You can be a utilitarian and claim that while A is $p$-responsible for E, the downstream consequences of containing or restraining A are worse, and that we therefore should not do so (Gregg, 2021). Or you can be a virtue ethicist and claim that virtues should be considered when assessing A’s $p$-responsibility, since someone’s virtues reflect prior probabilities of their A-ing.

The pragmatic position will possibly get us some of the way. A developer with an intention to produce E (with a small, non-zero probability) is more likely to produce E (with a small, non-zero probability) than a developer without this intention, all things being equal. This would hold, for example, if the developer was able to test E in the process of model selection. However, all things *are not equal*, and it will in practice be impossible to decide *a priori* which developer is more likely to develop a neural network bringing about E. The developer with no or only good intentions may, for all we know, sample an unlucky set of initial parameter values. The pragmatic position will thus not help us introduce accountability without compromising the entire field of artificial intelligence research and development. If regulation of artificial intelligence was based on probabilistic prevention strategies, there would as of now be no reason to hold one developer more accountable than another.

The pragmatic position is also agnostic about whether or not a $p$-responsibility gap exists. If $p$-responsibility solely turns on forward-looking measures to estimate risk, and not on retrospective responsibility assignment for harm done, the $p$-responsibility gap comes down to the risk profiles of humans and neural networks. The exact differences are an empirical question, but it seems clear that in practice both humans and neural networks introduce risks in some contexts. On this view, there is no decisive $p$-responsibility gap in practice, only different ways in which risk can manifest and be managed. The critical question is whether forward-looking measures that suffice for neural networks are also sufficient for humans, whose social and moral practices evolved partly to compensate for the lack of reliable risk estimation. If this is correct, one implication is that $p$-responsibility may be institutionally easier to assign to machines than to humans, since the relevant risk estimates can be empirically tested and iteratively improved. Yet, this also means that machines could be treated more leniently than humans with respect to retrospective blame, which may diverge from established norms of accountability. The pragmatic challenge, therefore, is not whether machines can in principle be subject to $p$-responsibility, but whether equalizing our standards for humans and machines distorts our practices of responsibility in ways that undermine the legitimacy of those standards. This indicates that the existence of a $p$-responsibility gap is not merely an empirical question about performance profiles, but also a normative one about the coherence of our responsibility practices.

In sum, the pragmatic position does not really let us side-step the responsibility dilemma induced by The All-or-Nothing Assumption. So far we have had no reason, however, to think we are faced with a responsibility gap – or a $p$-responsibility gap. We have shown that the gap question turns on unforeseeability – and the extent to which humans in general and neural network developers specifically are exempt from negligence – but are the negligence profiles of humans in general and neural network developers specifically qualitatively different or not? We now turn to this question, inspired by Fischer’s reply to Mele’s Zygote.

## Fischer’s Reply to Mele

In his compatibilist response to Mele, Fischer (Fischer, 2011) considers three alternative scenarios in which the zygote could be produced in more traditional ways:

1. John and Mary have intercourse, without the intention of pregnancy.
2. John and Mary have intercourse with the intention of pregnancy.
3. John and Mary have intercourse at time $t$ with the intention to trigger Ernie’s action A 30 years down the line.

Fischer argues that John and Mary are *not* morally responsible, even in F3, for Ernie’s A-ing. He appeals to common intuition for this. Since F3 is not relevantly different from Mele’s Zygote, Fischer argues, Diana (in Mele’s thought experiment) is *also* not morally responsible for Ernie’s A-ing. Given our discussion above it should be clear that our intuition is that F3 turns on John and Mary’s ability to foresee A. This is why the cognitive capacity of John and Mary – or Diana – is morally relevant. In practice, of course, Ernie’s A-ing is not foreseeable to John and Mary.

Note, however, how John and Mary are clearly acting with bad intentions in F3, even if their actions will not have the intended consequences. One reason to assign John and Mary $p$-responsibility is that equipped with a better theory of how they could cause Ernie to A, bringing about E, John and Mary would likely do so. So some form of punishment or quarantining can be justified in order to prevent them from doing so in the future (if there is a non-zero probability they acquire the relevant knowledge to improve the chances of a succesful experiment).

Mele’s example, in our view, is one where the ‘designer’ is $p$-responsible. This is because Ernie’s A-ing *is* foreseeable *to Diana*. To see this, consider a slippery slope running the opposite direction from F1–F3.

1. Diana composed Ernie’s zygote, bit for bit, with the intention to trigger Ernie’s action A 30 years down the line.
2. Diana planted a chip in Ernie at age 0, with the intention to trigger Ernie’s action A later in life.
3. Diana planted a chip in Ernie at age 30, with the intention to trigger Ernie’s action A a few months later.
4. Diana took control of Ernie’s brain in the moment, causing him to A.

Clearly, we want to hold Diana responsible in the case of M4. It seems the only difference between M4 and M1 is the risk of things going wrong over the course of time from intervention to end result. We want to argue that Diana’s or John’s or Mary’s intention increases the risk of Ernie’s A-ing. This is in itself reason to consider them $p$-responsible. Whether Ernie ends up A-ing 30 years down the line is of less relevance. Consider now:

1. Diana, with all good intentions, trains a neural network that after 30 days of all happy end users, As.
2. Diana trains a neural network at time $t$ with the intention to trigger the network to A.
3. Diana composes a neural network, weight for weight, so that it will A at time $t$.

We have now listed 10 Mele-like scenarios. In scenarios F3, M1–M4, S2, and S3, the actors exhibit bad intentions. In M1–M4 and S3, the agents manipulate the target more or less directly. Mele and Fischer disagree about M1, but how should we think about S1? This is the scenario we are in our motivating examples, Examples 1 and 2. Given empirical facts about neural networks, Diana has little control over the neural network she trains. Is putting the neural network to use reckless and negligent *in light of this* uncertainty? Neither Mele nor Fischer would hold Diana responsible for a neural network she cannot foresee the decisions of, it seems, but maybe some responsibility theorists would.

## Negligence

Most moral responsibility theorists would say that ‘people are responsible for the foreseen consequences of their actions’ (Raz, 2010), again seemingly vindicating Diana for developing the neural network in S1. But what if the actions are negligent? The standard definition of negligence – the failure to exercise the care of an ordinarily prudent and careful person, or the failure to behave with the level of care that someone of ordinary prudence would have exercised under the same circumstances – relies on peer estimates of what ordinary practice is. We have argued above that whether developers can be held responsible for neural network inferences turns on negligence, which in turn turns on whether we can decide which consequences were foreseeable. Let us flesh out the last step a bit.

Training or distributing a neural network can, on this view, be a negligent act if the developer fails to exercise the care of an ordinarily careful developer, but this means negligence turns on community standards. Past practice, however, is not a good yardstick for a developing technology. It is simply not clear whether the artificial intelligence community can meaningfully set its own standard for negligence. We leave this problem–call it The Negligence Community Standards for Emerging Technologies Problem–open for now, but assume that developing neural networks and putting them to use is sufficiently high-risk to be considered a potentially negligent action. Now, which consequences of such action should developers be responsible for?

One response will be to say developers should be responsible only for the foreseeable consequences, exercising prudence. However, we have already seen that it may not be possible to decide which consequences were foreseeable – or, at least, that this is an empirical, case-by-case question. So what can we do?

In the 1961 Privy Council decision, the court said that

[I]f it would be wrong that a man should be held liable for damage unpredictable by a reasonable man […] equally it would be wrong that he should escape liability […] if he foresaw or could reasonably foresee the intervening events which led to its being done […] Thus foreseeability becomes the effective test.

If we follow this line of reasoning, a neural network developer such as Diana is responsible only for the network’s *predictable* predictions, ruling out outlier (small, non-zero probability) events. However, this view is contested. Goodhart (1930), for example, discusses the Palsgraf case, in which a railway guard in assisting a passenger boarding a train knocked a package from his arms. The package turned out to contain fireworks–a fact of which the guard was ignorant–and an explosion followed. The concussion knocked over some scales standing a considerable distance away, and in falling they injured the plaintiff, a woman, who was an intending passenger. In this case, the jury was split, the majority holding that the guard was not liable, but the minority holding that ‘a negligent wrongdoer is liable for all the consequences of his act whether he could have foreseen them or not.’ From this minority view, we could distill a rationale for assigning responsibility to Diana in S1:

If we consider a developer’s training a neural network and putting it to use–with potential impact on the lives of others–an act of potentially negligent wrong-doing (Premise 1), and we accept that a negligent wrongdoer is liable for at least some of the consequences of their act whether they could have foreseen them or not (Premise 2), the developer is responsible for at least some of the consequences of all its predictions.

The majority view, i.e., what most courts would decide, is often expressed by the following principle:

**Unforeseeable Type of Harm** A person who causes injury to another is not liable if the type of harm does not foreseeably flow from the negligent act.

Where does this leave us? On the majority view, our reasoning will be: If Diana is only responsible in S1 if the A-ing of the neural network was foreseeable, the litmus test becomes whether the A-ing was foreseeable or not. Since this is generally not decidable, by $f_1--f_5$, the majority view is infeasible, leaving us with only two options:

(i) We can give up on accountability, leaving AI systems involving neural networks unregulated.
(ii) Accepting the minority view, i.e., full developer responsibility.

Both (i) and (ii) would of course present serious challenges to artificial intelligence research and development. The dilemma is similar to the dilemma in the Palsgraf case. It seems that unless we adopt a more pragmatic stance, i.e., $p$-responsibility instead of responsibility – there is no way out of the All-or-Nothing Assumption, even for humans. Clearly, the majority view is the majority view, because the minority view would make anyone liable for any unintended consequence of their actions, making us very sensitive to bad luck.

## Concluding Remarks

We have discussed whether developers of neural networks are responsible for the (possibly unintended) actions of these networks. Given empirical facts about neural networks, we derived a dilemma: Either developers must be responsible for all such actions or none at all. This seems to introduce a responsibility gap Matthias (2004), but we argue *it does not*: The situation is exactly the same as for humans. We are nevertheless left with two unpleasant alternatives: full developer responsibility or no responsibility at all. The first (the Palsgraf minority view) would scare off the developers, the second (the majority view) would scare off end users. It seems responding to neural networks from a more pragmatic stance, in terms of risk management or what we have called $p$-responsibility, presents us with a bit more wiggle room.

## Data Availability

Not applicable (no data has been used in this research) Contact details (must contain all author’ names, affiliations and contact details).

## Footnotes

1 Or, in the words of Burrell (2016): ‘Deep Learning models that prove useful […] possess a degree of unavoidable complexity.’

2 See, e.g., Matthias’s discussion of the NASA Pathfinder system (Matthias, 2004).

3 Alfred Mele’s Zygote Argument concerns whether the creation (Ernie) bears responsibility, whereas our main concern is whether the creator (the developer) bears responsibility. These are flip-sides of the same argument in the binary case in which either the creation or the creator bears responsibility. In the developer case, multiple parties may be responsible: developers, organizations, individual users, regulators and policymakers. The Zygote Argument, which concerns the binary case, may still be relevant. If the Zygote Argument is similar to our case in relevant respects, and leads us to think that in specific contexts, creators, not creations, are to blame, then we may tentatively blame developers citing the ways in which their case is similar to the Zygote case. We can of course also generalize the Zygote Argument to include organizations, individual users, regulators and policymakers.

4 There is a rich discussion as to whether the Zygote argument achieves Mele’s purpose, but we will sidestep that discussion here.

5 In 2018, the Stack Overflow Annual Developer Survey showed that 3/4 of developers feel responsible for neural networks they train.
